# Specimen retrieval during elective laparoscopic cholecystectomy: is it safe not to use a retrieval bag?

**DOI:** 10.1186/s12893-016-0181-y

**Published:** 2016-09-19

**Authors:** Muhamed Hamid Majid, Babak Meshkat, Haseeb Kohar, Sherif El Masry

**Affiliations:** 1 Beaumont Hospital, Beaumont road, Dublin 9, Ireland; 2 129 Verdemont, Snugborrough road, Blanchardstown, Dublin 15 Ireland; 3 Our Lady of Lourdes Hospital, Drogheda, Ireland

## Abstract

**Background:**

Since the introduction of laparoscopic surgery for gallbladder disease different types of retrieval devices have been used to extract the gallbladder from the peritoneal cavity. These devises infer additional costs and may lead to associated risks and complications. We aimed to evaluate the safety of gallbladder retrieval without the use of a retrieval device.

**Methods:**

A prospective study was conducted across two teaching hospitals in the Republic of Ireland from July 2010–2013. Patients undergoing planed elective day case laparoscopic cholecystectomy in the two institutions were included in the study. Data were collected on patient demographics, the use of a bag, any need for extension of fascial incision, any unexpected over night stay, any 30-day post operative complications and presence of port site hernia within 1 year surgery.

**Results:**

There were 373 planned elective day case laparoscopic cholecystectomy performed during the study period. A bag was not used to retrieve the gallbladder in 41 % (*n* = 152) patients. A retrieval bag was used in the majority of patients (71 %) who required over night stay due to pain. Overall wound infection rate was low (2.4 %), with 57 % of those being in patients where no retrieval bag was used. An increase incision in the fascia was required in 9.7 % of patients. The majority of these were in patients in whom a retrieval bag was used (75 %). At 1 year follow up, there were no recorded cases of port site hernia for the no retrieval bag group and two (0.9 %) cases of umbilical port site hernias in the group where retrieval bag was used.

**Conclusion:**

In cases of elective uncomplicated laparoscopic cholecystectomy for radiologically confirmed benign disease there was no benefit in using a retrieval bag. Furthermore, not using a bag was associated with less need for increasing the size of the fascial incision thereby reducing post operative pain and risk of port site hernia.

## Background

Laparoscopic cholecystectomy remains one of the most common surgical procedures performed worldwide [1]. In the developed world 90 % of cholecystectomies are completed laparoscopicaly. Since the introduction of laparoscopic surgery for gallbladder disease different types of retrieval devices have been used to extract the gallbladder from the peritoneal cavity. These ranged from simple non-powdered gloves to several types of commercially produced bags [2, 3]. The use of retrieval devices have been advocated for several reasons, including prevention of wound infection and avoidance of port site metastasis [4–10]. In laparoscopic cholecystectomy, their use is thought to provide the further benefit of reducing the risk of stone spillage into the peritoneal cavity. However, the use of retrieval bags can make removal of the specimen more difficult, requiring enlargement of the port site incision and potential risk of abdominal organ damage during bag insertion and retrieval [11, 12].

In cases of elective laparoscopic cholecystectomy, there is rarely an ongoing inflammatory process which obviates the risk of wound infection during specimen retrieval. Furthermore, when the gall bladder is dissected free without spillage of its contents, there is no further benefit in placing it in a retrieval bag and in fact further manipulation by trying to do so may lead to stone spillage. With these factors in mind, we aimed to evaluate the safety of gallbladder retrieval without the use of a retrieval device.

## Methods

A prospective audit of ongoing practice of three consultant surgeons across two teaching hospitals in the Republic of Ireland from July 2010–2013 was conducted. All participating surgeons had more than 10 years experience as consultants and together over 5000 laparoscopic cholecystectomies performed. It was standard practice for two of the surgeons to use a commercially available retrieval bag (EndoCatch ®, Covidien, USA) for all cases when removing the gallbladder from the abdominal cavity, while one surgeons standard practice was to use a retrieval bag only if there was spillage of bile or stones from the gallbladder.

Pre-operative diagnosis of gall stones was confirmed using biliary ultrasound scan in all cases. Patients undergoing planed elective day case laparoscopic cholecystectomy in the two institutions were included in the study. Those who had bile or stone spillage during the procedure were excluded as this necessitated the use of a retrieval bag for all surgeons. The patients were selected to undergo their surgery with the participating surgeons through the standard referral pathway and all had a consultation with the operating consultant or a member of their team prior to surgery. All patients gave written informed consent to undergo laparoscopic cholecystectomy. The audit of practice conducted for this study was registered with the hospital audit board. Only patients with body mass index of <30 kg/m^2^ were eligible for admission for elective day-case laparoscopic cholecystectomy and were included in the study.

All patients underwent standard four port laparoscopic cholecystectomy with initial para-umbilical camera port inserted using Hasson technique, a further 10 mm epigastric port and two 5 mm ports on right side were inserted under direct vision. Once the gallbladder was dissected free, it was retrieved through the umbilical port (either using a retrieval bag or not) under direct laparoscopic visualisation by moving the camera into the epigastric port. The gallbladder was not aspirated, and stones were left intact within the gallbladder. In cases where the specimen was too large for extraction the facial incision was increased to facilitate extraction. Standard prophylactic antibiotics included single dose of 1.2 g Co-Amoxiclav at the time of induction, with patients who were known penicillin allergic receiving 1.5 g Cefuroxime instead. There was no protocol in place for pre-operative bathing and all patients were admitted to the hospital on the day of their procedure. Umbilical port site closure was with polyglyconate absorb-able sutures (0-Maxon). Skin incisions were closed with either metal skin clips or a subcuticular suture using 4-0 monocryl.

The operative protocol, antibiotics use, pre- and post operative management of patients was the same in both institutions as its was the same medical team (surgical and anaesthetic) working across both sites. Furthermore both institutions were under same management, allowing for homogeneous care across the two institutions.

Follow up for all patients was standardized, with each arranged to attend their general practitioner (GP) 1 week after surgery. Patients were given written information on the signs and symptoms of wound infection, and given a contact number for urgent out patient assessment in cases where a wound infection was suspected. Similar written information was provided to GPs with instructions to contact the research team in cases where post operative wound infection was suspected.

A superficial wound infection was defined as a skin or subcutaneous infection requiring antibiotics. A deep wound infection was defined as an infection requiring drainage and exploration of the wound.

A 1-year follow-up appointment was provided to all patients to assess for any delayed complications of surgery. All patients who attended the hospital after 1 year underwent a thorough clinical exam to look for a port site hernia. If there was any doubt about the clinical findings the patient was sent for an abdominal wall ultrasound to look for the presence of a hernia.

Data were collected on patient demographics, the use of a bag, any need for extension of fascial incision and any unexpected over night stay. The collected data were maintained in an institutional review board-approved database.

Categorical data were compared using the two-tailed Fisher exact test and a *p*-value less than 0.05 was considered significant.

## Results

There were 373 planned elective day case laparoscopic cholecystectomy performed during the study period, the majority of whom were women (76.4 %, *n* = 285). A bag was not used to retrieve the gallbladder in 41 % (*n* = 152) patients compared to 69 % (*n* = 221) in whom a retrieval bag was used. Retrieval bag rupture was recorded in three patients (1.4 %). Table 1 outlines the demographics data of the patients.

**Table 1:** Demographics

	All patients	No retrieval bag used	Retrieval bag used
Number of patients	373	152	221
Age (years)	Mean = 52 (range 21–71)	Mean = 53 (range 23–67)	Mean = 52 (range 21–71)
Male	24 % (*n* = 88)	23 % (*n* = 35)	24 % (*n* = 53)
Female	76 % (*n* = 285)	77 % (*n* = 117)	76 % (*n* = 168)
Follow up after 1 year	51.5 % (*n* = 192)	54 % (*n* = 82)	50 % (*n* = 110)
Missing 1 year follow up data	48.5 % (*n* = 181)	46 % (*n* = 70)	50 % (*n* = 110)
Benign histology	100 %	100 %	100 %

Overall 1.9 % (*n* = 7) of patients required unexpected over night stay due to pain, 71 % (*n* = 5) of these were patients in whom a retrieval bag was used and the remaining 29 % (*n* = 2) in patient in whom retrieval bag was not used. There were no other recorded cases of unexpected over night stay and all patients who stayed over night for analgesic control were discharged on post operative day one.

There were nine (2.4 %) recorded wound infections during the study, with the vast majority being superficial wound infections (78 %, *n* = 7). Of the patients presenting with superficial wound infections, 57 % (*n* = 4) were in patients in whom retrieval bag was not used and the remaining 43 % (*n* = 3) in patients where a retrieval bag was used. All superficial wound infections were treated with oral antibiotics and required no further intervention. There were two recorded deep wound infections, one in each group (retrieval bag used and retrieval bag not used). Both patients required drainage of wound collection.

An increase incision in the fascia was required in 9.7 % (*n* = 36) of patients. The majority of these were in patients in whom a retrieval bag was used (75 %, *n* = 27).

One year follow up data was collected for 51.5 % (*n* = 192) of patients with the remaining 48.5 % (*n* = 181) not returning to their 1 year follow up appointment. The post operative 1 year follow up attendance between the two groups was similar at 54 % (*n* = 82/152) and 50 % (*n* = 110/221) for the no retrieval bag used and retrieval bag used groups respectively. At 1 year follow up, there were no recorded cases of port site hernia for the no retrieval bag group and two (0.9 %) cases of umbilical port site hernias in the group where retrieval bag was used. Both of which were diagnosed on clinical basis and required no imaging.

Histological examination showed no evidence of malignancy in any of the removed specimens.

Table 2 outlines the comparative results of length of stay (LOS), wound infection, need for increasing fascial incision and port site hernias between the two groups.

**Table 2:** Comparing variables between the two groups

Variables	No Bag Used	Bag Used	Relative Risk	Odds Ratio	*P* Value
LOS >24 h	1.32 %	2.26 %	0.58	0.54	1.000
Sup Wound Inf	2.63 %	1.35 %	1.97	1.94	1.000
Deep Wound Inf	0.66 %	0.45 %	1.47	1.47	1.000
Port site Hernia	0 %	1.81 %	0.27	0.26	0.40
Fascia Cutting	5.92 %	12.22 %	0.48	0.45	0.19

## Discussion

There remains controversy regarding the optimal retrieval device for laparoscopic cholecystectomy. In this small study, using no retrieval device in cases of intact gall bladder in uncomplicated cases showed no statistically significant difference in the post operative LOS, wound infection rates, need for fascial incision extension and port site hernia rates at 1 year follow up. Although there was no statistical significant difference between the two groups, it was noted that post operative pain, need for increasing fascial incision and occurrence of port site hernias were more common in patients where a retrieval bag was used.

The incidence of port site infection is quite low in laparoscopic cholecystectomy, however it remains unclear as to whether port site infection is due to contamination with the contents of the gallbladder or from bacteria present on the patients skin. A study performed by R. Harling et al. [4] showed that in the majority of cases cultures grown from port site infections were skin rather than biliary organisms. As patients with intra-operative bile leak were excluded in this study, it is likely that organisms responsible for wound infections would be similar to those reported by Hurling et al. However it is acknowledged that no swabs were taken in this study to assess whether port site infections occurring in patients who had their gallbladder removed without the use of bag were skin or biliary organisms. Despite the exclusion of patients who had bile leakage, it was noted that wound infection rates were almost twice as common in patients in whom a retrieval device was not used. While this value did not reach statistical significance in the current study, the noted trend would raise the possibility that mare contact of the intact gall bladder may increase the risk of wound infection. However the patients in this study were not controlled for material used for skin closure which may have been a more important factor in development of wound infections. While there is lacking data on the difference of wound healing after laparoscopic cholecystectomy when using skin clips rather than subcuticular sutures. In other studies sutured closure of wounds are suggested to be superior to stapled closure [13–15].

All patients included in this study were planned to undergo day case laparoscopic cholecystectomy. The unexpected over night stay rate was 1.9 % which is within the acceptable range and comparable to other institutions [16]. While no statistically significant difference was noted between the two groups with regards to need for over night stay, more patients having their gallbladder retrieved using a bag were kept over night than those in whom a bag was not used. Similarly the patients in whom a bag was used required more frequent extension of the fascial incision and it was only in this group that incisional hernias were recorded. These findings would suggest that the use of bag may more frequently lead to the need for increasing fascial incision, thereby increasing post operative pain and risk of incisional hernia. However the numbers did not reach statistical significance. The size and weight of the gallbladder in each case was also not recorded, which may well be a more important factor for the need to extend the fascial incision than the use of a retrieval bag.

The 1 year post operative follow up in this study was just over 50 % which further limits the interpretation of rates of insicional hernia recorded. However the recorded rate of 1.8 % in the cohort in which a retrieval bag was used is comparable to reported rates in other studies [17].

An often raised concern for why a retrieval bag should be used is to reduce the risk of cutaneous seeding of potential malignant cells if there is histological gallbladder malignancy. Previous studies have shown the incidence of malignancy at the time of cholecystectomy to range from 0.44 to 1.28 % [18–20]. There were no recorded cases of gallbladder malignancy in this study. With adequate pre-operative imaging and normal gallbladder appearance intraoperatively, this risk should be extremely low, but can never be eliminated.

## Conclusion

While many retrieval devices have been developed and are frequently used across the world in laparoscopic cholecystectomy, there is no consensus on the optimal retrieval method. This study is the first to evaluate whether in fact any retrieval device is necessary in cases of uncomplicated elective laparoscopic cholecystectomy. The findings suggest that where there is radiological confirmed benign disease there is no benefit in using a retrieval bag. Furthermore, not using a bag may reduce the need for increasing the size of the fascial incision thereby reducing post operative pain and risk of port site hernia.

## Abbreviations

GP: General practitioner
LOS: Length of stay

## References

[1] Litwin DE, Cahan MA (2008). Laparoscopic cholecystectomy. Surg Clin North Am.

[2] Holme JB, Mortensen FV (2005). A powder-free surgical glove bag for retraction of the gallbladder during laparoscopic cholecystectomy. Surg Laparosc Endosc Percutan Tech.

[3] Patton JT, Jorgensen J, Imrie CW (1997). Specimen retrieval in laparoscopic cholecystectomy British. J Surg.

[4] Harling R, Morejani N, Perry C, MacGowan AP, Thompson MH (2000). A prospective, randomised trial of prophylactic antibiotics versus bag extraction in the prophylaxis of wound infection in laparoscopic cholecystectomy. Ann R Coll Surg Engl.

[5] Samel S, Post S, Martell J, Becker H (1997). Clostridial gas gangrene of the abdominal wall after laparoscopic Cholecystectomy J. Laparoendosc. Adv Surg Tech A.

[6] Al-Awami SM, Al-Breiki H, Abdul-Khader AS, Twum-Danso K, Grant C, Wosomu L (1191). Wound infection following biliary surgery. A prospective study. Int Surgery.

[7] Pezet D, Fondrinier E, Rotman N (1992). Parietal seeding of carcinoma of the gallbladder after laparoscopic chole- cystectomy. Br J Surg.

[8] Drouard F, Delamarre J, Capron JP (1991). Cutaneous seeding of gallbladder cancer after laparoscopic cholescystectomy. N Engl J Med.

[9] Fong Y, Brennan MF, Turnbull A, Colt DG, Blumgart LH (1993). Gallbladder cancer discovered during laparoscopic surgery. Potential for iatrogenic tumor dissemination. Arch Surg.

[10] Silk YN, Douglas HO, Nava HR, Driscoll DL, Tartarian G (1989). Carcinoma of the gallbladder. The Roswell Park experience. Ann Surg.

[11] Upadhyaya M, Sundararajan LS, Woodward MN (2011). Dangerous deliveries: lessons learned during retroperitoneal specimen retrieval. J Pediatr Surg.

[12] Schellpfeffer MA (2011). A Novel laparoscopic tissue retrieval device. JSLS.

[13] Chughtai T, Chen LQ, Salasidis G, Nguyen D, Tchervenkov C, Morin JF (2000). Clips versus suture technique: is there a difference?. Can J Cardiol.

[14] Bash SL, Rochon ML, Quinones JN, Coassolo KM, Rust OA, Smulian JC (2010). Randomized Controlled trial of wound complication rates of subcuticular suture vs staples for skin closure at cesarean delivery. Am J Obstet Gynecol.

[15] Mackeen AD, Khalifeh A, Fleisher J, Vogell A, Han C, Sendechi J, Pettker C, Leiby BE, Baxter JK, Sfakianaki A, Berghella V (2014). Suture compared with staple skin closure after cesarean delivery: a randomized controlled trial. Obstet Gynecol.

[16] Al-Qahtani HH, Alam MK, Asalamah S, Akeely M, Ibrar M (2015). Day-case laparoscopic cholecystectomy. Saudi Med J.

[17] Alptekin H, Yilmaz H, Acar F, Kafali ME, Sahin M (2012). Incisional hernia rate may increase after singe-port chlecystectomy. J Laparoendosc Adv Surg Tech A.

[18] Jacobi CA, Keller H, Monig S, Said S (1995). Implantation metastasis following laparoscopy. Surg Endosc.

[19] Razzetta F, Borgonovo G, Cagnazzo A, Bianci C, Mattioli FP (1997). Laparoscopic cholecystectomy and gallbladder cancer: a diagnostic and therapeutic dilemma. Eur J Surg Oncol.

[20] Copher JC, Rogers JJ, Dalton ML (1195). Trocar-site metastasis following laparoscopic cholecystectomy for unsuspected carcinoma of gallbladder. Surg Endosc.

